# Ventriculosternal Shunt for the Treatment of Idiopathic Normal Pressure Hydrocephalus: A Case Report

**DOI:** 10.3389/fsurg.2021.607417

**Published:** 2021-08-23

**Authors:** Xinxia Guo, Abdul Malik Popal, Zhoule Zhu, Chengwei Cai, Jingquan Lin, Hongjie Jiang, Zhe Zheng, Jianmin Zhang, Anwen Shao, Junming Zhu

**Affiliations:** Department of Neurosurgery, The Second Affiliated Hospital, School of Medicine, Zhejiang University, Hangzhou, China

**Keywords:** cerebrospinal fluid shunting, ventriculosternal shunting, normal pressure hydrocephalus, intraosseous absorption, case

## Abstract

**Background:** Conventional corticospinal fluid (CSF) diversion surgery for idiopathic normal pressure hydrocephalus (iNPH) includes ventriculoperitoneal shunt and ventriculoatrial shunt. Ventriculosternal (VS) shunt may be considered if both the abdominal cavity and atrium are not feasible.

**Methods:** A 76-year-old woman was admitted to our hospital with gait disturbance and urinary incontinence for 2 years, and the condition aggravated in the last 1 month. Based on clinical assessment and imaging findings, the patient was diagnosed with iNPH, with surgical indications. She was on peritoneal dialysis for chronic renal failure, and a cardiac Doppler echocardiogram showed enlargement of the left atrium and decreased diastolic function of the left ventricle. Due to these conditions, we chose the sternum as the vessel for CSF absorption and performed VS shunt.

**Results:** No swelling, exudation, and effusion were found in the suprasternal fossa. Gait disturbance and urinary incontinence improved significantly immediately and 1 week after surgery, respectively. No shunt-related complication was reported at 16 months follow-up.

**Conclusion:** This case demonstrated VS shunting as a feasible and alternative for the management of hydrocephalus.

## Introduction

Idiopathic normal pressure hydrocephalus (iNPH) is a common neurodegenerative disease among the elderly characterized by gait disturbance, cognitive decline, and urinary incontinence (1). In the treatment of iNPH, ventriculoperitoneal (VP) shunt, and ventriculoatrial (VA) shunt are the most commonly performed procedures to divert corticospinal fluid (CSF), both of which result in significant improvements of functional outcomes (2, 3). However, in certain patients who have peritonitis, adhesion, and cardiac insufficiency, there seems to be no standard alternative surgical method to be considered. Recently, Ming Woo et al. reported a case about ventriculosternal (VS) shunt for the management of hydrocephalus, which provided a potential option for these patients (4). In the past, the sternum has been used for volume resuscitation in an emergency situation. Macnab et al. showed that the sternal gravity infusion flow could reach 80 ml/min,

and the injector injection flow could reach 150 ml/min by testing the sternum as an alternative site for fluid resuscitation (5). Tubbs et al. demonstrated that the sternum is a plausible receptacle for CSF diversion by an experimental work (6). In this work, we report a case of iNPH for whom VS shunt was performed, and we further illustrate the feasibility of VS shunt.

## Case Presentation

**Figure 1:**
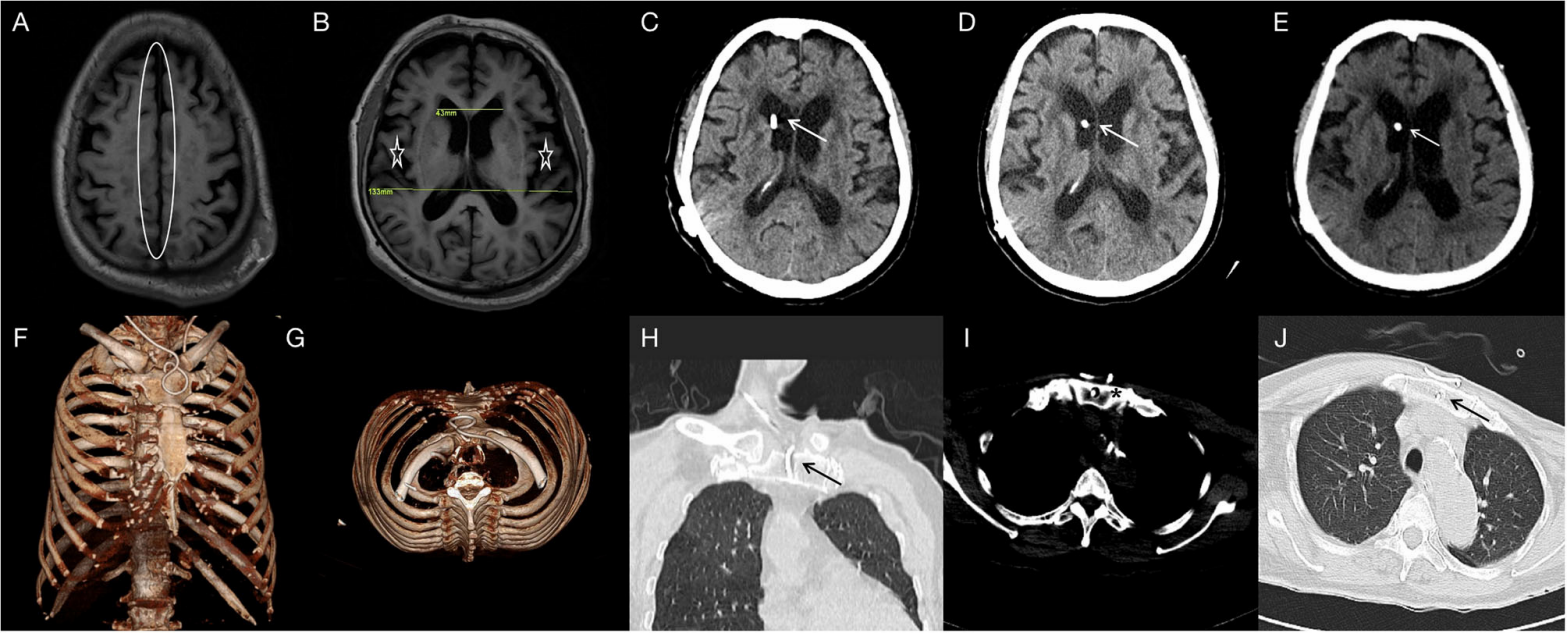
T1FLAIR **(A,B)** brain MRI showing the narrowed sulcal spaces at the vertex (oval) and the enlarged ventricles of the brain and lateral cracks(asterisk) (DESH); Evans Index was 0.32. Two-month CT-**(C)**, 6-month CT-**(D)** and 16-month CT-**(E)** brain scan showing the catheter in position and the hydrocephalus is relieved. One-week three-dimensional reconstructed CT- **(F,G)** scan of the thorax showing the distal catheter *in situ*. Two-month CT- **(H,I)** and 16-month CT-**(J)** thorax scan showing the sternum is intact and the distal catheter *in situ*.

A 76-year-old woman was admitted to our hospital complaining of progressive gait disturbance, continuous urinary incontinence, and gradual decline in cognitive function for 2 years. She was diagnosed with Type 2 diabetes for 5 years and hypertension for 26 years, and she was on peritoneal dialysis for chronic renal failure (Primary IgA nephropathy) for 13 months. On admission, a B-ultrasound of the urinary system showed that both the kidneys were atrophied. The serum creatinine and urea values were 505 umol/L and 11.53 md/dL, respectively. Magnetic resonance imaging (MRI) showed disproportionately enlarged subarachnoid space hydrocephalus (7, 8) (Figures 1A,B) and dilated cerebral ventricles and cisterns, with no apparent obstacle (Figure 1B). Evans Index was 0.32 (Figure 1B). The iNPH grading scale (iNPHGS) (9) showed 2 points in cognitive impairment, 3 points in gait assessment, and 3 points in the urinary disturbance domain (Table 1). The mini-mental state examination (MMSE) (10)score was 23 of 30. The CSF of the tap test of the patient (11), which rated with the 10-m up test and 5-meter up & go test before, and 8 and 72 h after 30 ml of CSF removal (CSF pressure was 160 mmHg), showed improvement (Table 2). Laboratory studies of CFS were normal. A diagnosis of iNPH was made. Surgical treatment was indicated in this patient. However, she was on peritoneal dialysis for chronic renal failure, and a cardiac Doppler echocardiogram showed enlargement of the left atrium and decreased diastolic function of the left ventricle. Due to these conditions, and after careful evaluation, we chose the sternum as the vessel for CSF absorption. Informed consent was obtained from the patient and her family.

**Table 1:** iNPH grading scale.

**Grade**	**Definition**	**Before surgery**	**After surgery (one week)**
**Cognitive impairment**			
0	Normal		√
1	Complaints of amnesia or inattention but no objective memory and attentional impairment	√	
2	Existence of amnesia or inattention but no disorientation of time and place		
3	Existence of disorientation of time and place but the conversation is possible		
4	Disorientation for the situation or meaningful conversation impossible		
**Gait disturbance**			
0	Normal		
1	complaints of dizziness of drift and dysplasia but no objective gait disturbance		√
2	unstable but independent gait		
3	walking with any support	√	
4	walking not possible		
**Urinary disturbance**			
0	Normal		
1	pollakiuria or urinary urgency		√
2	occasional urinary incontinence (1–3 or more times per week but less than once per day)		
3	continuous urinary incontinence (1 or more times per day)	√	
4	bladder function is almost or entirely deficient		

*Positive outcomes:*

*(1) For gait, a positive outcome was defined as >1-point improvement in the gait section of iNPHGS*.

*(2) For cognition, a positive outcome was defined as >1-point improvement in the cognition section of iNPHGS*.

*(3) For urinary incontinence, a positive outcome was defined as >1-point improvement in the urinary incontinence section of iNPHGS*.

**Table 2:** The CSF tap test results.

**Test**	**Before the CSF Tap Test**	**After 8 h**	**Improvement**	**After 72 h**	**Improvement**
5 m Up & Go test	32 s, 32 steps	34 s, 26 steps	−6.3*, 18.8%	21 s, 21 steps	34.4, 34.4%
10 m up test	19 s, 30 steps	14 s, 23 steps	26.3, 23.3%	13 s, 21 steps	31.6, 30.0%

*Positive outcomes:*

*(1) 10 m up test: One parameter was improved more than 20%, or two parameters were improved more than 10%*.

*(2) 5 m Up & Go test: A more than 10% rate improvement after the CSF Tap Test*.

(3) *The reason for this calculation result may be that the step distance of the patient is widened and the number of steps decreases during the gait test, or it may be caused by artificial measurement errors.

## Surgical Procedure

**Figure 2:**
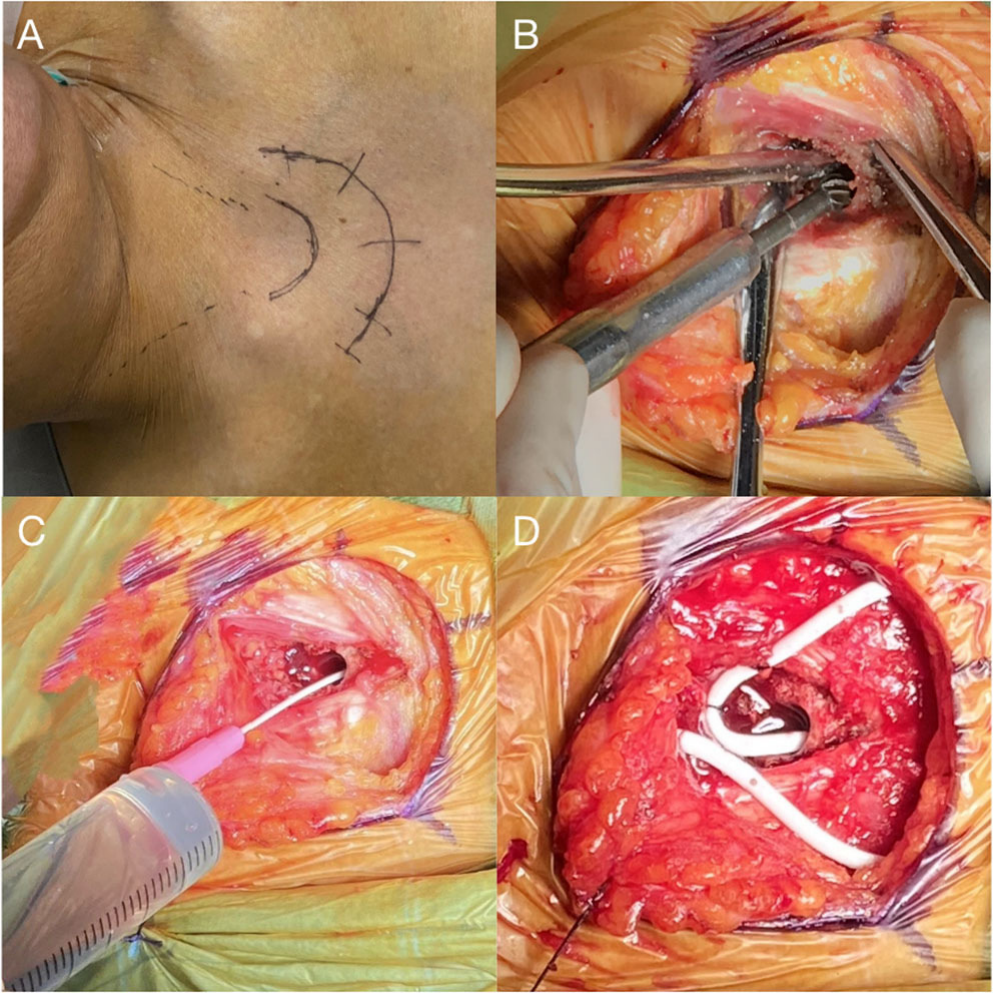
**(A)** An arc skin incision for a right-sided shunt is marked. **(B)** Drilling of a 3 cm long tunnel parallel to the long axis of the sternum at its midline. **(C)** Indwelling needle placement to confirm the length of the tunnel and intraosseous absorption of 10 ml saline. **(D)** Placement of sternal catheter with a loop fixed at the sternum tendon of the sternocleidomastoid muscle.

The patient was given general anesthesia and took the supine position with a slightly raised right shoulder and back. After skin preparation and draping, an arc skin incision was made at the sternal notch (Figure 2A), and then subcutaneous tissues were separated until the anterior edge of the bilateral sternocleidomastoid muscle and the superior border of the sternum were exposed. Next, a tunnel, approximately 3 cm long and parallel to the sternum, was drilled through the sternal manubrium by a high-speed drill (Figure 2B). An indwelling needle containing 10 mL of saline was used to confirm the length of the tunnel and test the intraosseous absorbability (10 mL injected in 3 min; Figure 2C). A subcutaneous tunnel was made from the scalp incision to the sternal notch incision with a metal strip. An adjustable pressure antisiphon shunting catheter (ProGAV 2.0, Aesculap), parallel to the sagittal line, was then inserted through the right frontal bone burr hole at the Kocher's point. The adjustable gravity valve was connected to the ventricular catheter, pressure preadjusted to 120 mm H_2_O. The distal catheter was placed into the sternal manubrium, and a reserved loop was fixed at the sternum tendon of the sternocleidomastoid muscle (Figure 2D). After confirming that the shunt tube was unobstructed, the tunnel entrance was sealed with bone wax to prevent CSF leak. Tissue and skin of each

incision were sutured in layers with absorbable 2-0 polyglactin-910 sutures.

## Postoperative Course

**Figure 3:**
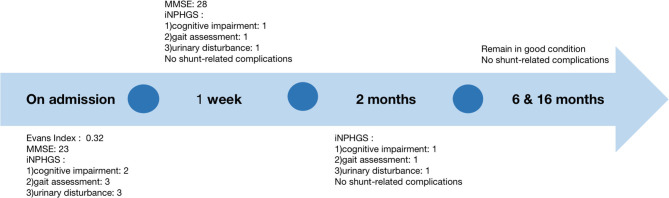
A timeline of the follow-up of the patient.

The patient recovered well without fever, headache, and had no shunt-related complications after surgery. No swelling, exudation, and effusion were found in the suprasternal fossa. Laboratory blood studies showed no signs of associated infection. Gait disturbance and urinary incontinence improved significantly 2 days and 1 week after surgery, respectively. The three-dimensional reconstructed thoracic CT scan suggested that the distal catheter was located in an intraosseous tunnel, and there was no complication, such as manubrium sternal fracture, pneumothorax, or mediastinal gas (Figures 1F,G). The MMSE score improved to 28 of 30, and the scores of all three domains of the iNPHGS were rated at 1 point (Table 1). At the 2-month follow-up, the scores of all three domains of the iNPHGS remained the same. The CT brain scan showed the ventricle size did not dramatically change (12), and the ventricular catheter was in place (Figure 1C). **T**he CT thorax scan showed sternum was intact, and there were no shunt-related complications(Figures 1H,I). The patient remained in good condition and was free of shunt-related complications at the 6-month and 16-month follow-up (Figures 1D,E,J). The episode of care is organized as a timeline in Figure 3. At the 16-month postoperative follow-up, the patient did not feel any inconvenience caused by this surgical method.

## Discussion

The CFS diversion surgery has four basic components, namely: a proximal catheter, a reservoir, a valve, and a distal catheter (13, 14). In general, the proximal catheter drains CSF from one of the two lateral cerebral ventricles. The reservoir provides an access to obtain CSF samples and for intracranial pressure monitoring. The valve ensures unidirectional CSF flow into the distal catheter. Traditionally, the distal catheter is tunneled subcutaneously and terminates in a cavity, which can absorb the shunted CSF (13, 15). There are variations in the placement of distal catheters such as peritoneal, atrial, sternum, etc., to adapt to different conditions of patients (15).

In this study, we reported a typical case of iNPH, who was not suitable for routine VP or VA shunt because of cardiac insufficiency and renal failure; so we introduced an alternative called VS shunt. The patient had significant improvement and no obvious complications during the 16-month follow-up.

Based on the literature, the sternum is ideal for distal catheter placement for the following reasons: (1) Its location is adequately superficial for shunt implantation (4, 16); (2) Its proximity to the skull reduces the risk of pulling and displacement caused by the overlength of the shunt catheter (4, 16); (3) It retains more red bone marrow than the tibia and humerus in adults, allowing better absorption of CSF (4, 16); (4) internal mammary vein and azygos vein, allowing adequate drainage of CSF (4, 17); (5) the post operation fasting time of VS shunt is only 6 h, to avoid patients suffer from thirst, sore throat, and hunger, and also to accelerate the recovery of normal gastrointestinal function; (6) compared with VA shunt, VS shunt avoids serious complications such as atrial thrombosis, arrhythmia or endocarditis, especially in the elderly with cardiac dysfunction.

According to existing researches, the sternum also has been supported as a feasible and alternative drainage vessel in physiology, animal experiments, and clinical cases. Physiologically, the bone marrow cavity contains abundant cavernous venous sinuses, which can communicate with the systemic circulation through the central tube, nourishing vein, and guiding vein (16). Fluids or drugs can quickly enter the circulatory system *via* the bone marrow cavity. A previous work has demonstrated that the sternum can absorb flow rates of up to 150 mL/min (5), and sternal intraosseous infusion applied as a rapid, safe, and effective vascular access when venous punctures failed in emergency treatment in adults (18). According to the above researches, CSF could be rapidly absorbed in the bone marrow cavity.

Ventriculosternal shunt was first proposed by Tubbs et al. based on an exploratory study (6), in which he first injected up to 30 L of water continuously into the manubrium sternum of five fresh human cadavers within 1 h, and no significant fluid overflows, edema, or fluid accumulation were observed in the chest or abdomen. The team then injected saline into the sternum of living rhesus monkeys for 24 h, and the MRI showed no extravascular fluid accumulation. To verify the feasibility of VS shunt in large animals, they performed the procedure on two adult pigs. Two weeks after surgery, the conditions of both the pigs were stable without any sign of infection. There was also no evidence of osteomyelitis or CSF exudation in the autopsy. Based on these findings, Tubbs et al. argued that the manubrium sternum was an ideal “CSF container”.

The first successful application of VS shunt in the treatment of hydrocephalus was reported by Ming Woo et al. In this case, the physiology and cognition of the patient were recovered to a certain extent and suffered no complications 3 years after the procedure (4). As for details of VS shunt procedure, Ming Woo et al. suggested that the length of the tunnel on the sternal manubrium should be controlled within 4 cm to avoid damage to the brachiocephalic artery by accidental puncture of the posterior sternal cortex or entry into the superior mediastinum (4). The surgeon should be aware that long-term CSF immersion may lead to osteomalacia and pathological fractures. In the later stage, attention should be paid to postoperative scar formation, especially for young patients with a family history of skin scars or dark-skinned patients. Another concern is that long-term CSF immersion may lead to osteomalacia and pathological fractures. They also mentioned the possible contraindications of VS shunt, including tracheotomy, sternal fracture or history of sternotomy, osteogenic insufficiency or severe osteoporosis, local infection, cardiac shunt from right to left, and history of shunt glomerulonephritis (4).

According to our study, we also concluded the following technical notes on VS shunt surgical procedure: (1) thoracotomy design: with the upper sternal fossa as the center, a 2/3 circular incision was formed to expose the upper sternal fossa; (2) sternal tunnel: parallel to the long axis of the sternal manubrium when drilling, with a depth of <4 cm; (3) test the absorption rate of CSF: ensure that 10 mL of saline can be absorbed within 3 min. We found that it could be challenging to test intraosseous absorbability with saline due to bleeding intraoperatively, and for further study, except the potential risks and complications mentioned above by Ming Woo et al., we propose that VS shunt, as with conventional shunt methods, can also develop complications like shunt-related idiopathic intracranial hypertension (IIH), infection, over drainage, and so on (19–21).

Otherwise, ventriculovesical (VV) shunt and ventriculo-cholecystic (VC) shunt have been reported as alternative surgeries for CSF diversion (22–25). However, Shahul Hameed et al. suggested that VV shunt should not be recommended as an alternative to peritoneum or atrium, because of the existing disadvantages, which are: it is a difficult surgical technique, fluid electrolytes depletion, and recurrent urinary tract infection and calculus formation (23). According to available reports, VC shunt is generally performed on pediatric patients. Duncan Henderson et al. reported that the VC shunt survived for 1 year in two children, who had preoperative external ventricular drain (EVD) outputs of 8 and 10 ml/h, respectively. And one child failed at day four, who had preoperative EVD of 30–35 ml/h (22). Hence, the EVD was suggested to evaluate per hour of CSF production pre-VC shunt (22), which is an invasive operation increasing the physical burden and surgical risks to patients. The reflux of bile causing an aseptic meningitis and irritation of the brain stem also have been reported after post-VC shunt by Bernstein et al. (24). Relatively, VS shunt was more feasible for this patient.

According to our knowledge, only two cases of hydrocephalus treated with VS shunt have been reported, including our case study. The limitation is that the period of follow-up time in our case is not long enough to support VS shunt as a long-term feasible alternative, although the outcome was considerable at 16-month follow-up. The potential risks and possible complications of this novel procedure have not been fully investigated as well. Despite, this the VS shunt may be a potential option for CSF diversion in the future.

## Conclusion

The authors demonstrate the feasibility and efficacy of VS shunt for the management of iNPH in this case report. VS shunt may be a valuable option when other conventional CSF diversion procedures are ruled out.

## Data Availability Statement

The raw data supporting the conclusions of this article will be made available by the authors, without undue reservation.

## Ethics Statement

The studies involving human participants were reviewed and approved by Ethics Committee of the Second Affiliated Hospital of Zhejiang University, School of Medicine. The patients/participants provided their written informed consent to participate in this study. Written informed consent was obtained from the individual(s) for the publication of any potentially identifiable images or data included in this article.

## Author Contributions

All authors contributed toward surgery records, drafting, and revising the article.

## Conflict of Interest

The authors declare that the research was conducted in the absence of any commercial or financial relationships that could be construed as a potential conflict of interest.

## Publisher's Note

All claims expressed in this article are solely those of the authors and do not necessarily represent those of their affiliated organizations, or those of the publisher, the editors and the reviewers. Any product that may be evaluated in this article, or claim that may be made by its manufacturer, is not guaranteed or endorsed by the publisher.
